# Child-Directed Speech Is Semantically Constrained: Evidence From Metaphoricity and Concreteness

**DOI:** 10.1162/OPMI.a.387

**Published:** 2026-08-21

**Authors:** Liudmyla Feurstein, Iulia M. Comsa, Reinhard Furrer, Kim Gfeller, Paul Widmer, Srini Narayanan, Sabine Stoll

**Affiliations:** Institute for the Interdisciplinary Study of Language Evolution (ISLE), University of Zurich, Zurich, Switzerland; Google Zurich, Zurich, Switzerland; Department of Mathematical Modeling and Machine Learning, University of Zurich, Zurich, Switzerland

**Keywords:** child-directed speech, metaphor, concreteness, language acquisition, caregiver input

## Abstract

Child-directed speech (CDS) is widely theorized to provide a structured scaffold for early language acquisition, yet the semantic properties of this register remain insufficiently quantified. In particular, it remains unclear how figurative language and semantic grounding interact within caregiver input to stabilize early form–meaning mappings. We present a large-scale computational analysis of metaphoricity and lexical concreteness in a naturalistic corpus of CDS, evaluated against a size-matched baseline of adult-directed speech (ADS). Using a token-level metaphor detection model and human-indexed semantic norms, we test two primary accounts: the reduced metaphoricity hypothesis (H1), which predicts a systematic reduction of nonliteral expressions in caregiver input, and the lexical concreteness hypothesis (H2), which predicts an intensified reliance on perceptually grounded vocabulary. Cross-register comparisons revealed robust support for both accounts: CDS contained a significantly lower density of metaphorical expressions and a higher baseline of both continuous and categorical lexical concreteness than ADS. Longitudinal mixed-effects models further showed that these semantic constraints undergo gradual, age-dependent tuning. As children mature, caregivers appear to relax these constraints incrementally, diversifying their figurative repertoire and reducing overall vocabulary concreteness over time. Taken together, these findings suggest that the semantic structure of caregiver speech functions as a dynamic, responsive learning environment. By initially favoring concrete, transparent, and literal forms, CDS may reduce interpretive ambiguity during early phases of reference anchoring before gradually shifting toward the more abstract and flexible lexical patterns characteristic of adult speech.

## INTRODUCTION

One of the defining features of human language is its productivity: speakers routinely generate and understand expressions they have never previously encountered. Much research on language acquisition has historically focused on syntactic creativity—the capacity to combine a finite set of rules into an unbounded set of utterances (Chomsky, 1980; Hockett, 1960; Martinet, 1960). This computational problem is frequently framed in terms of the poverty of the stimulus, whereby children appear to derive abstract, complex grammatical knowledge from highly variable and limited input (Chomsky, 1957, 1965).

Yet this emphasis on structural compositionality has often overlooked a complementary and equally formidable challenge: semantic learning. Unlike syntactic acquisition, semantic acquisition may involve not only deriving abstract patterns from limited input, but also navigating a dense, highly variable, and often ambiguous input environment—what might be described as an *overabundance of the stimulus*—in which words routinely shift meaning across contexts and are frequently deployed nonliterally (Fujita, 2021; Gentner & Asmuth, 2019; Lakoff & Johnson, 1980).

A central component of this interpretive challenge is lexical creativity, defined as the flexible extension of word meanings across conceptual boundaries, including metaphorical use. Speakers routinely employ words such as *grasp*, *see*, or *cold* in ways that extend far beyond their literal, physical denotations (Brochhagen et al., 2023; Shutova, 2015). These extensions are not peripheral or poetic anomalies but are fundamental to natural language: they allow speakers to recycle familiar lexical material to express novel, complex meanings and to construct abstract representations out of concrete experience (Clark, 1993; Gaskins et al., 2024; Gaskins & Rundblad, 2025; Gentner, 1983; Gentner & Boroditsky, 2001).

From a developmental perspective, however, lexical creativity poses a significant barrier to early comprehension. Children must learn not only the foundational, literal references of words but also the conceptual mechanics governing how those meanings extend across semantic domains. Empirical work demonstrates that children’s comprehension and production of metaphorical language emerge gradually over development, remaining fragile throughout early childhood (Di Paola et al., 2020; Gaskins et al., 2024; Gaskins & Rundblad, 2025; Pouscoulous & Tomasello, 2020; Zhu et al., 2024). This developmental lag raises a fundamental question: how is the primary linguistic input structured to scaffold this interpretive capacity?

Child-directed speech (CDS) provides an ideal testing ground for investigating this structural scaffolding. CDS differs systematically from adult-directed speech (ADS) across prosodic, syntactic, and lexical dimensions (Fernald, 1985; Fernald et al., 1989; Rowe, 2012; Snow, 1972; Soderstrom, 2007). It is widely assumed that these systematic modifications facilitate language learning, although their precise functional pressures remain under debate (Kempe et al., 2024). While CDS may be structurally optimized for pedagogy, it simultaneously serves immediate interactional and pragmatic goals, such as regulating infant affect, maintaining attention, and maximizing communicative engagement (Jones et al., 2023; Odijk & Gillis, 2021; Schick et al., 2022; Zhou et al., 2024). Moreover, recent ethnographic work cautions that direct dyadic CDS may constitute only a partial component of children’s broader linguistic environments, further complicating its functional interpretation (Bunce et al., 2025; Schick & Stoll, 2025).

A commonly proposed but under-quantified property of the caregiver register is semantic simplification. Caregiver speech has repeatedly been shown to rely on restricted vocabularies, recurrent lexical frames, and structured repetition patterns that may facilitate acoustic segmentation and early lexical mapping (Hills, 2013; Lester et al., 2022; Mintz et al., 2002; Schwab & Lew-Williams, 2016). Together, these structural observations suggest that CDS provides a semantically constrained learning environment that minimizes cognitive load, yet large-scale quantitative evidence documenting how semantic constraints are structured remains limited.

The present study addresses this empirical gap by focusing on two core dimensions of semantic structure: metaphoricity and lexical concreteness. While these dimensions are often discussed independently in psycholinguistic literature, their structural interaction is central to understanding the mechanics of lexical creativity. Metaphorically extended meanings typically recruit concrete source domains to structure highly abstract target concepts, indicating a systematic relationship between figurative language and semantic grounding (Lakoff & Johnson, 1980). Examining both dimensions jointly within naturalistic datasets therefore allows for a more precise, multidimensional characterization of the spoken input available to developing learners.

Using large-scale naturalistic corpora of spoken dialogue, we evaluate the semantic architecture of caregiver input along two distinct empirical axes. First, we test the reduced metaphoricity hypothesis (H1), which predicts that CDS contains fewer metaphorical expressions than ADS. Second, we test the lexical concreteness hypothesis (H2), which predicts that CDS relies more heavily on perceptually grounded vocabulary (Gros-Louis et al., 2006; Hoff, 2006; Motamedi et al., 2024; Newport, 1977; Rowe, 2012; You et al., 2021).

Rather than treating developmental change as an independent theoretical domain, we evaluate age-related variations strictly as a supporting robustness check. Because children must ultimately scale their linguistic competence to reach adult-level proficiency, we expect caregiver input to exhibit a gradual, longitudinal relaxation of these semantic boundaries. Observing an age-dependent progression toward adult-directed baselines over time would support the interpretation that CDS operates as a dynamic, responsive linguistic environment.

Together, these analyses provide a rigorous quantitative characterization of the semantic structure of CDS and offer new insights into how the linguistic environment stabilizes early form–meaning mappings.

## METHODS

### Data

Language learning in infancy is fundamentally shaped by social interaction, as children acquire linguistic knowledge through engagement with multiple interlocutors in their environment (Bruner, 1983; Tomasello, 2003). Studying the creative properties of this input therefore benefits from methods that extend beyond laboratory experiments, which, while offering important experimental control, may not fully capture the dynamics of naturalistic interaction. To determine whether lexical creativity differs across spoken registers, it is essential to compare ecologically valid samples of language use.

Here, we used transcriptions of recordings of English-speaking children in their home environments as they engaged in everyday activities (Lieven et al., 2009; Theakston et al., 2001). The child-directed dataset included 14 longitudinal caregiver–child interaction corpora (child age range: 1;3–3;0), downsampled to a three-million-word sample. These recordings provide a rich source of conversational data, capturing spontaneous, naturalistic exchanges between caregivers and children.

For comparative purposes, we used a sample of adult conversation from the British National Corpus (BNC), size-matched to the CDS corpus at three million words (BNC Consortium, 2007; You et al., 2021). The BNC is a monolingual British English corpus comprising approximately 100 million words. Its spoken component includes transcriptions of unscripted informal conversations, balanced for factors such as age, region, context, and social class, as well as recordings from more formal settings such as meetings (Burnard, 2007; McEnery & Hardie, 2011).

### Metaphor Annotation

#### Automatic Metaphor Detection With MelBERT.

Recent advances in natural language processing have demonstrated that the extraction of lexical extensions is feasible by integrating multiple features to predict creativity. For example, the combination of visual and linguistic embeddings has revealed that metaphor and cross-domain vocabulary projection are inherently encoded in distributional semantic spaces across languages (Shutova et al., 2017). Additional factors that enhance automatic metaphor detection include both categorical and sensory features, such as concreteness scores, topic distributions, and parts of speech; incorporating these features consistently improves model performance (Tong et al., 2021). Beyond metaphor detection, emerging models can generate human-like lexical extensions, such as concept mappings between source and target domains (Ge et al., 2022).

To annotate metaphorical words in our corpora, we employed the MelBERT model, a novel metaphor-detection system based on the Metaphor Identification Procedure (MIP) (Choi et al., 2021). MIP and its elaborated MIPVU protocol identify a word as metaphorical when its contextual meaning contrasts with a more basic meaning that remains available in comparison (Steen et al., 2010). MelBERT (Metaphor-aware late interaction over BERT) operates as a classification model that determines whether a target word within a sentence is metaphorical. The model achieves state-of-the-art performance across several benchmark datasets (Choi et al., 2021).

MelBERT is thus trained to perform a binary classification task: for each target word in a sentence, classify whether the word is metaphorical. To do this, MelBERT relies on two key ideas:

1. The first is the Metaphor Identification Procedure (Steen et al., 2010), which proposes that a word or phrase is metaphorical when its contextual meaning differs from its literal meaning.
2. The second is the older and related idea of selectional preference violation (SPV) (Choi et al., 2021; Wilks, 1978). Here, the goal is to quantify how much a target word’s meaning departs from the meanings of the associated words in the sentence.

As an example from the paper describing MelBERT (Choi et al., 2021), consider the sentence “Don’t twist my words.” The contextual meaning of *twist*—“to distort the intended meaning”—differs from its literal meaning, “to form into a bent, curling, or distorted shape.” Thus, under the MIP protocol, it is labeled as a metaphor. In addition, *twist* violates selectional preferences, as its literal meaning is unusual in the context of the surrounding words. In the model, MelBERT uses a Siamese architecture that takes two inputs: (1) the sentence *S* containing the target word *w*_*t*_, and (2) the target word *w*_*t*_ in isolation. MelBERT independently encodes *S* and *w*_*t*_ into embedding vectors. In accordance with MIP, MelBERT uses the contextualized and isolated representations of *w*_*t*_ to distinguish between its contextual and literal meanings. To incorporate SPV, MelBERT compares the sentence embedding vector and the contextualized target-word embedding vector, identifying how much the surrounding words mismatch the target word. The model combines these signals to classify whether a word is metaphorical.

MelBERT was selected because it combines pretrained contextual representations with theory-informed metaphor classification and strong benchmark performance.^1^

#### Human Annotation and Reliability.

To assess the degree of agreement between human annotators, as well as between human judgment and MelBERT annotation, we extracted two samples of our corpora, each containing 5,000 words. For our reliability analyses, we used the same text samples for both MelBERT and human annotation. The human raters were two research assistants who annotated the samples from both corpora. Annotation conventions were based on an abridged version of the MIPVU procedure (Steen et al., 2010). A final consensus annotation was established for each sample after interrater reliability had been calculated. The annotation procedure, along with all annotated samples, is available at https://github.com/lingresearchers/LexCreativity.

We used Cohen’s kappa (*κ*) as a measure of agreement between annotators (McHugh, 2012). For CDS, interrater reliability was *κ* = 0.33 (interpreted as fair agreement (Cohen, 1960; Landis & Koch, 1977)), with raw agreement of 95%. For ADS, *κ* = 0.60 (moderate agreement), raw agreement = 93.3%. Agreement between the consensus annotations and MelBERT annotations showed similar patterns: for CDS, *κ* = 0.53 (moderate), raw agreement = 96%; and for ADS, *κ* = 0.56 (moderate), raw agreement = 94%.

We acknowledge that metaphor identification is inherently difficult. Interrater reliability was modest for CDS (*κ* = 0.33), reflecting the ambiguity of metaphor in short, context-limited utterances typical of caregiver–child interaction. In such data, utterances are often fragmentary, lack broader discourse context, and contain underspecified referents, all of which reduce agreement on whether a given word is used metaphorically.

Importantly, low *κ* values in this setting are not unexpected. Metaphor identification—even under standardized procedures such as MIPVU—has been shown to yield only moderate agreement in naturalistic spoken data, particularly when annotations are performed at the word level and without extended discourse context (Shutova, 2015; Steen et al., 2010). Moreover, *κ* is sensitive to class imbalance: because the majority of words are used literally, high raw agreement can co-occur with relatively low *κ* values (McHugh, 2012).

To address this limitation, we rely on multiple safeguards. First, analyses are conducted on large-scale corpora, where aggregate patterns are robust to individual annotation decisions. Second, we compare across registers using the same annotation procedure, ensuring that any systematic bias applies equally across conditions. Third, our main findings are convergent across independent measures (metaphoricity and concreteness), reducing the likelihood that results are driven by annotation noise alone.

These considerations suggest that, while individual metaphor judgments are uncertain, the observed differences between CDS and ADS reflect reliable distributional tendencies rather than annotation artifacts.

#### Context Window Selection.

MelBERT annotations can be adjusted by modifying the window size used during computation. We initially ran the model with a 100-token window. However, mean length of utterance (MLU) differs substantially across registers: MLU is only 3.9 tokens in CDS and 9.4 tokens in ADS. Because such short utterances render a 100-token window inappropriate—particularly for child-directed and spoken language—we also ran the model using a 3-token window to better align the annotation context with the actual utterance lengths.

We also calculated Pearson correlation coefficients to compare the results of MelBERT computations based on 3-token and 100-token context windows across registers. For metaphorical word types, the correlation between the two window sizes was lower in CDS, *r* = .85, *p* < .001, than in ADS, *r* = .95, *p* < .001. This pattern suggests that window choice affected CDS annotations more strongly than ADS annotations, supporting our decision to report the smaller-window analyses.

### Measures

#### Metaphor Frequency.

After completing MelBERT annotation for the CDS and ADS corpora, we preprocessed the texts by performing tokenization and word segmentation. After identifying all word tokens, we conducted our statistical analyses. We calculated the percentage of metaphorical words relative to the total number of words in each text or speech sample and computed their means across registers. We performed analyses for both word types and word tokens to control for the higher repetitiveness characteristic of CDS. As noted above, we also computed statistics for both window sizes (3 and 100 tokens) for the initial comparison of metaphor percentages. In all subsequent analyses, we relied exclusively on the output from the MelBERT window-size-3 computation.

As a supplementary descriptive analysis, we examined the lexical concentration of metaphor annotations in each spoken register. For CDS and ADS, we extracted all word tokens classified as metaphorical by MelBERT and calculated the frequency distribution of metaphorical word types. We then identified the 20 most frequent metaphorical types in each register and computed their proportional coverage relative to the total number of metaphorical tokens. For ADS, this analysis was restricted to the matched spoken files used in the window-size comparison.

#### Concreteness.

In addition to metaphor labeling, we analyzed the concreteness scores of the word types present in CDS versus ADS, motivated by the fact that children’s early lexicon consists largely of concrete terms (Bergelson & Swingley, 2013; Buccino et al., 2019; Nencheva et al., 2023). A higher proportion of concrete concepts in CDS would support the idea that this register is adapted for early vocabulary acquisition.

Concreteness ratings are a widely used approach to operationalize the difference between concrete and abstract words (Reijnierse et al., 2019). Studies that collect concreteness ratings typically define the distinction in terms of direct sensory experience. For example, words such as ‘banana’ are rated as relatively concrete, while words such as ‘idea’ are rated as relatively abstract (Brysbaert et al., 2014).

To calculate the average concreteness scores for our corpora, as well as to analyze the concreteness ratings of the word types, we used two datasets of human concreteness ratings (Brysbaert et al., 2014; Snefjella et al., 2019).

The resource created by Brysbaert et al. (2014) consists of 39,954 English words and two-word expressions rated for concreteness on a scale from 1 to 5, where 1 denotes the most abstract concepts and 5 denotes the most concrete, physically grounded ones (Brysbaert et al., 2014). Participants were instructed that concrete concepts “refer to things or actions in reality, which you can experience directly through one of the five senses,” whereas abstract concepts “refer to meanings that cannot be experienced directly” (Winter & Srinivasan, 2022). For illustration, “wood” has a rating of 4.85, while “counterargument” has a rating of 2.17, and “conventionalism”, 1.18 (Alnafesah et al., 2020).

The second source of concreteness ratings (Snefjella et al., 2019) was derived using the “SocialSent” software package (and its key algorithm, SentProp, which makes use of word embeddings; Hamilton et al., 2016). The authors provide computed concreteness scores for 37,058 word types. The ratings in this dataset, which we used as a validation measure, are categorical: the scores are abstract (−1), neutral (0) or concrete (1).

The two concreteness resources showed strong agreement on their shared lexical items. Brysbaert et al.’s resource (Brysbaert et al., 2014) contained 39,954 entries, while the Snefjella and colleagues dataset (Snefjella et al., 2019) contained 37,058 entries. Of these, 37,057 entries overlapped. The remaining 2,897 Brysbaert-only entries consisted almost entirely of multi-word or hyphenated expressions, whereas our corpus-level analyses were conducted on tokenized single-word forms. Across the 37,057 overlapping entries, the two rating systems were highly correlated: Pearson’s *r*(37,055) = .907, *p* < .001, Spearman’s *ρ* = .917, *p* < .001, and Kendall’s *τ* = .791, *p* < .001. After preprocessing and lexical normalization, the rated corpus vocabulary fell within this shared overlap, yielding identical item coverage across the two resources for both CDS and ADS.

Our concreteness datasets have several limitations. Not all word types in our corpora are covered, as the available word lists are restricted to relatively frequent items. Consequently, a subset of tokens and types did not receive concreteness scores. However, through targeted preprocessing (e.g., removal of extraneous characters and contractions, lexical normalization, and correction of part-of-speech tags for lemmatization), we achieved high token-level coverage in both corpora. In CDS, 2,533,679 word tokens received ratings, corresponding to 94.78% coverage; in ADS, 2,891,879 word tokens received ratings, corresponding to 94.32% coverage. Type-level coverage was lower: 7,067 CDS word types received ratings, corresponding to 67.43% coverage, while 16,304 ADS word types received ratings, corresponding to 61.05% coverage.

In addition, previous work has noted that some concreteness ratings in the (Brysbaert et al., 2014) dataset can be problematic or counterintuitive (Beigman Klebanov et al., 2015). Nevertheless, multiple studies have validated this dataset as a reliable resource for concreteness-related linguistic analyses (Martínez et al., 2024; Proos & Aigro, 2024). Given the large number of rating observations per word (at least 25), and the overall high consistency across datasets, we can reasonably assume that any scoring anomalies cancel out in the aggregate.

### Statistical Analyses

#### Group Comparisons.

To compare metaphor usage between CDS and ADS, we conducted one-way ANOVAs with register as a two-level factor.

For concreteness measures, the ratings from Brysbaert et al. (2014) were treated as continuous scores. We calculated mean concreteness separately at the token and type levels and compared CDS and ADS using separate one-way ANOVAs for each measure. In contrast, the ratings from Snefjella et al. (2019) were categorical; for this dataset, we calculated concrete-to-abstract ratios for word tokens and the percentages of concrete, neutral, and abstract word types in each register.

#### Developmental Analyses.

We examined the relationship between children’s age and the percentage of metaphorical words found in the CDS corpus. To control for individual differences among children and their linguistic environments, we built a mixed-effects model with both random intercepts and random slopes. This model predicts the session-level percentage of metaphors (as annotated by MelBERT) used in the speech of caregivers as a function of the child’s age over time. To ensure mathematical consistency across our developmental frameworks, the mixed-effects model tracks sessions *i* nested within individual caregiver–child dyads *j* as follows:

$$\text{Prop}\_\text{met}\_\text{types}\_{\text{CDS}}_{ij}=\beta_{0}+\beta_{1}{\text{Age}}_{ij}+u_{0j}+u_{1j}{\text{Age}}_{ij}+\epsilon_{ij}$$

where:

- Prop_met_types_CDS_*ij*_ represents the proportion of metaphorical types in the child-directed input for session *i* within dyad *j*,
- *β*_0_ is the fixed intercept,
- *β*_1_ is the fixed slope associated with child age,
- *u*_0*j*_ is the random intercept for dyad *j*,
- *u*_1*j*_ is the random slope associated with child age for dyad *j*,
- *ϵ*_*ij*_ is the residual error term.

The random intercept (*u*_0*j*_) and random slope (*u*_1*j*_) account for longitudinal variability across individual dyads, and *ϵ*_*ij*_ represents the residual session-level error. Identical formulas were used to predict nonliteral caregiver word tokens rather than types.

To study the relationship between the child’s age and the concreteness of words in CDS, we fitted separate linear mixed-effects models using an identical nested notation structure. The dependent variables were mean token-level and mean type-level concreteness in CDS, modeled separately, with child age as the fixed effect and random intercepts included to account for the nesting of sessions within the 14 dyads. This model is specified as follows:

$$\text{Conc}\_{\text{CDS}}_{ij}^{\left(k\right)}=\beta_{0}+\beta_{1}{\text{Age}}_{ij}+u_{0j}+\epsilon_{ij}$$

where:

- $\text{Conc}\_{\text{CDS}}_{ij}^{\left(k\right)}$ represents the mean concreteness of CDS in session *i* for dyad *j*, computed either at the token level or the type level, indexed by *k*,
- *β*_0_ is the fixed intercept,
- *β*_1_ is the fixed slope associated with child age,
- Age_*ij*_ represents the age of the target child in session *i* for dyad *j*,
- *u*_0*j*_ is the random intercept for caregiver–child dyad *j*,
- *ϵ*_*ij*_ is the residual error term.

Random slopes for age were not included in the concreteness specification due to the limited number of unique dyads (*n* = 14) and convergence issues observed during initial testing.

All statistical analyses were conducted in either R (R Core Team, 2020) or Python (Van Rossum & Drake, 2009) using the following packages: “NLTK” (Bird et al., 2009), “scipy” (McKinney, 2010), “sklearn” (Pedregosa et al., 2011), “LexicalRichness” (Shen, 2022), “statsmodels” (Seabold & Perktold, 2010), and “itertools” (Python Software Foundation, 2026). All scripts and datasets are available at https://github.com/lingresearchers/LexCreativity.

## RESULTS

### Metaphoricity

Consistent with H1, the proportion of metaphorical words per speech sample was significantly lower in CDS than in ADS across MelBERT window sizes and across both token- and type-based measures. The mean percentages for each register, calculated from the matched files used in the window-size comparison, are presented in Table 1 and visualized in Figure 1.

**Table 1.** Mean percentage of metaphorical words per speech sample relative to the total number of words across spoken registers.

**Corpus**	**Metaphorical words**			
	**Window size 100**		**Window size 3**	
	**Tokens**	**Types**	**Tokens**	**Types**
CDS	5.77%	10.46%	5.55%	9.79%
ADS	11.86%	19.18%	8.98%	14.59%

**Figure 1.**
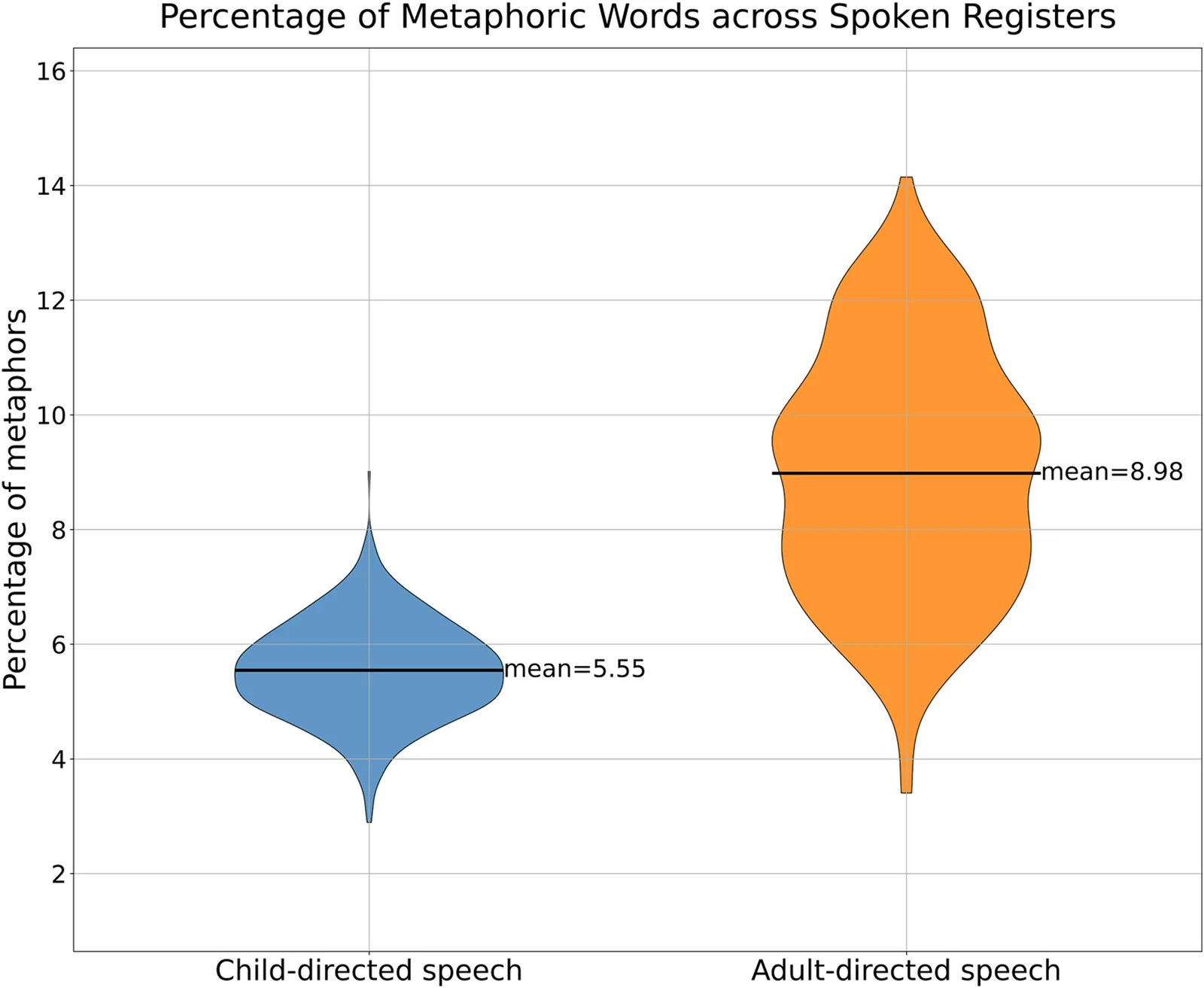
Violin plots of the percentages of metaphorical tokens per speech sample in the CDS and ADS corpora. MelBERT computations based on window size of 3 tokens. Whiskers show the whole range of data distribution. Black solid lines indicate the means.

The one-way ANOVAs (Table 2) revealed significant differences in the percentages of metaphorical words between CDS and ADS across all analyses. This pattern held for both MelBERT window sizes and for both token- and type-based measures. Across all comparisons, CDS contained lower proportions of metaphorical words than ADS. For the 100-token window, CDS had lower metaphorical-token percentages (*M* = 5.77%) than ADS (*M* = 11.86%), and lower metaphorical-type percentages (*M* = 10.46%) than ADS (*M* = 19.18%). The same pattern was observed for the 3-token window, with lower metaphorical-token percentages in CDS (*M* = 5.55%) than ADS (*M* = 8.98%), and lower metaphorical-type percentages in CDS (*M* = 9.79%) than ADS (*M* = 14.59%).

**Table 2.** ANOVA results for differences in the percentages of metaphorical words between the ADS and CDS registers.

**Metric**	**Window size 100**		**Window size 3**	
	**Tokens**	**Types**	**Tokens**	**Types**
**CDS *M* (%)**	5.77	10.46	5.55	9.79
**ADS *M* (%)**	11.86	19.18	8.98	14.59
***F***	1973.010	1462.325	1201.023	1029.077
***df***	1, 868	1, 868	1, 870	1, 870
***p***	<.001	<.001	<.001	<.001

The distribution of metaphor annotations was highly concentrated in both registers, but especially in CDS (Table 3). The 20 most frequent metaphorical types accounted for 70.98% of all metaphorical tokens in CDS and 54.12% in the matched ADS sample used for the window-size comparison. This indicates that CDS had a more compressed metaphorical vocabulary, with a smaller number of highly recurrent metaphorical types accounting for a larger share of all metaphor annotations.

**Table 3.** Lexical concentration of metaphor annotations across spoken registers.

**Corpus**	**Met. tokens**	**Unique met. types**	**Top-20 tokens**	**Top-20 coverage**
CDS	150,650	2,534	106,930	70.98%
ADS	261,228	5,858	141,375	54.12%

#### Developmental Trends in Metaphoricity.

Table 4 summarizes the model predicting the percentage of metaphorical word *types* in CDS from child age. Child age significantly predicted the percentage of metaphorical word types (*β* = .174, *p* = .021).

**Table 4.** Mixed-effects model results predicting the percentage of metaphorical word *types* in CDS from child age.

**Parameter**	**Coef.**	***SE***	***p***	**95% CI [0.025, 0.975]**
Intercept	9.856	0.129	<.001	[9.602, 10.109]
Age	0.174	0.075	.021	[0.026, 0.322]
Group Var	0.202	0.085		
Group × Age Cov	−0.008	0.032		
Age Var	0.039	0.025		

Table 5 summarizes the model output for predicting percentage of metaphorical word *tokens* in CDS from age. In contrast, child age did not significantly predict the percentage of metaphorical word tokens (*β* = .032, *p* = .542).

**Table 5.** Mixed-effects model results for predicting percentage of metaphorical word *tokens* in CDS from child age.

**Parameter**	**Coef.**	***SE***	***p***	**95% CI [0.025, 0.975]**
Intercept	5.569	0.121	<.001	[5.331, 5.806]
Age	0.032	0.052	.542	[−0.071, 0.135]
Group Var	0.192	0.118		
Group × Age Cov	−0.027	0.036		
Age Var	0.022	0.023		

Thus, metaphorical *types*, but not metaphorical *tokens*, increased with child age. This type–token dissociation suggests that developmental change in caregiver input is driven primarily by lexical diversification rather than by greater overall figurative density: caregivers introduce a broader range of metaphorically usable words as children mature, while maintaining a relatively stable rate of nonliteral token use.

### Concreteness

Both human concreteness datasets indicated that CDS was more concrete than the adult-directed conversational baseline. Using the human-rated continuous concreteness norms from Brysbaert et al. (2014), CDS showed higher mean concreteness than ADS at both the token and type levels. The continuous Brysbaert ratings are summarized in Table 6; the categorical SocialSent results are reported below.

**Table 6.** Continuous concreteness scores and coverage across spoken registers using Brysbaert et al.’s ratings.

**Register**	**Unit**	***M***	***N* rated**	**Coverage**
CDS	Tokens	2.81	2,533,679	94.78%
CDS	Types	3.63	7,067	67.43%
ADS	Tokens	2.61	2,891,879	94.32%
ADS	Types	3.18	16,304	61.05%

A one-way ANOVA revealed highly significant differences in continuous concreteness scores between CDS and ADS at both the token and type levels (Table 7). CDS was more concrete than ADS in both analyses, with a larger mean difference for word types than for word tokens.

**Table 7.** ANOVA results for differences in continuous concreteness scores between spoken registers.

**Level**	**CDS *M***	**ADS *M***	**MD**	***F***	***df***
Tokens	2.81	2.61	0.20	48952.69	1, 5425556
Types	3.63	3.18	0.45	925.74	1, 23369

*Note*. MD = mean difference, calculated as CDS minus ADS. Both tests were significant at *p* < .001.

Regarding the dataset with categorical concreteness ratings (Snefjella et al., 2019), we calculated the ratio of concrete to abstract word tokens for both CDS and ADS. The CDS corpus showed a higher concrete-to-abstract token ratio (0.77) than ADS (0.47), indicating a greater prevalence of concrete vocabulary in child-directed input.

We also examined semantic grounding at the level of word types by computing the percentages of unique concrete, neutral, and abstract word types across registers. Consistent with the token-based distributions, Table 8 shows that CDS contained a lower proportion of unique abstract words and a higher proportion of unique concrete words, confirming that child-directed input is lexically biased toward sensory-grounded concepts compared to the adult baseline.

**Table 8.** Percentages of categorical concreteness ratings assigned to word types across spoken registers.

**Register**	**Concrete %**	**Neutral %**	**Abstract %**
CDS	50.8%	38.1%	11.0%
ADS	32.7%	48.7%	18.7%

Figure 2 illustrates these cross-register differences for both concreteness rating datasets, using normalized values (means and ratios) for direct comparison.

**Figure 2.**
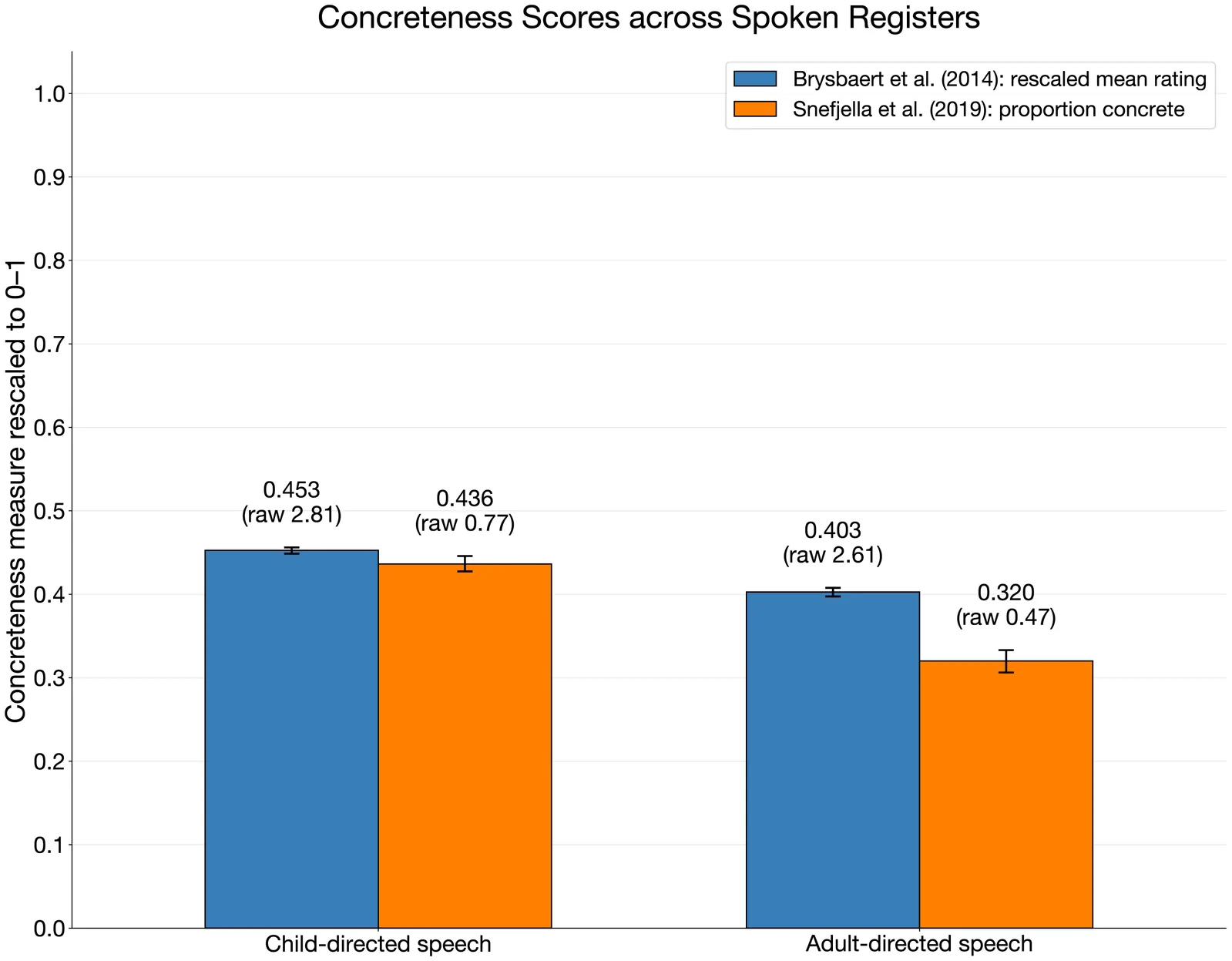
Normalized comparison of concreteness measures across spoken registers. Bars show Brysbaert mean concreteness scores and SocialSent concrete-to-abstract ratios, each normalized separately for visualization.

Together, the continuous and categorical analyses indicate that child-directed speech relies on a more concrete vocabulary base than adult-directed speech, supporting H2.

#### Developmental Trends in Concreteness.

Mixed-effects models predicting mean concreteness in CDS from child age revealed significant negative age effects at both the token and type levels (Table 9). Older children received slightly less concrete caregiver input, indicating a small but reliable age-dependent shift toward more abstract vocabulary over development.

**Table 9.** Mixed-effects model results predicting mean word concreteness in CDS from child age.

**Level**	**Parameter**	**Coef.**	***SE***	***p***	**95% CI**
Tokens	Intercept	2.804	.009	<.001	[2.787, 2.820]
	Age	−.014	.001	<.001	[−.017, −.011]
	Group Var	.001	.011		
Types	Intercept	3.295	.019	<.001	[3.256, 3.333]
	Age	−.030	.003	<.001	[−.037, −.024]
	Group Var	.005	.025		

Overall, these findings show that CDS is characterized by significantly higher average lexical concreteness than ADS, supporting H2. In addition, child age negatively predicted both token- and type-level concreteness in CDS, indicating a small but systematic shift toward more abstract vocabulary over time.

## GENERAL DISCUSSION

The present study provides evidence that child-directed speech (CDS) is semantically constrained along two key dimensions: it is significantly less metaphorical and substantially more concrete than adult-directed conversational registers. These findings align with foundational accounts proposing that early linguistic input prioritizes conceptual transparency and physical grounding during the initial stages of lexical acquisition (Ferguson, 1977; Newport, 1977; Snow, 1972). Sensory-grounded, concrete language may facilitate the real-time mapping between novel phonological forms and their real-world referents, whereas a reduced figurative load may limit semantic ambiguity while children are still establishing stable literal word meanings (Brysbaert et al., 2014; Gentner, 1982). By reducing the cognitive demands associated with parsing nonliteral meanings, CDS may provide a scaffolded communicative environment aligned with the developing child’s processing constraints.

Importantly, this semantic architecture may not arise solely from explicit pedagogical intent or conscious language-teaching strategies. Rather, it likely reflects broader interactional and pragmatic pressures, including maintaining children’s attention, regulating affect, coordinating joint attention, and simplifying dyadic communication in immediate, here-and-now social contexts (Fernald, 1985; Fernald et al., 1989; Soderstrom, 2007). At the same time, recent cross-cultural and ethnographic research cautions against treating intensive, highly modified one-on-one CDS as a universal baseline or strict prerequisite for language development. Caregiver modifications vary substantially across socio-economic and cultural communities; in many sociolinguistic contexts, direct child-directed speech may constitute only one component of children’s broader linguistic environments, which frequently include overheard adult conversations, multi-party interactions, and peer-to-peer communication (Bunce et al., 2025; Schick & Stoll, 2025; Shneidman & Goldin-Meadow, 2012). Therefore, the highly concrete and literal profile observed in our corpus should be understood as a robust feature of dyadic CDS, but one that operates within a diverse ecosystem of environmental inputs.

From a usage-based perspective, such distributions may facilitate early language acquisition by providing children with highly frequent, concrete, and contextually stable form–meaning mappings. Usage-based theories propose that children initially acquire language through repeated exposure to low-level, item-based patterns before gradually forming more abstract semantic and grammatical constructions (Lieven et al., 2003; Tomasello, 2003). Within this framework, relatively literal and low-ambiguity input may support early word segmentation and semantic stabilization prior to more flexible or figurative extensions. A higher density of metaphorical shifts or highly abstract reference early in development could make the task of isolating stable semantic cores more difficult, given the vast interpretative possibilities of an ungrounded signal (cf. Quine, 1960).

Furthermore, the reduced metaphorical density and increased concreteness observed in CDS may have important consequences for the development of lexical schemas. In usage-based accounts, abstract linguistic representations emerge gradually through bottom-up generalizations over repeated, deeply contextualized linguistic experiences rather than through pre-specified maturation schedules (Dąbrowska & Lieven, 2005; Hartmann et al., 2021). Frequent exposure to concrete and perceptually grounded lexical items may therefore strengthen the entrenchment of early semantic representations and facilitate later abstraction.

This structural property of CDS raises intriguing questions regarding *how* and *when* children transition from this heavily grounded baseline to adult-like figurative competence. As children age and their cognitive capacities expand, the linguistic input they encounter appears to undergo a gradual reduction in semantic scaffolding. The gradual introduction of abstract nouns and metaphorical expressions in later childhood may contribute to the alignment of the child’s semantic network with adult-directed norms. Crucially, these initially stabilized literal representations may provide an important conceptual baseline—or set of source domains—for the development of more flexible abstract reasoning and figurative interpretation later in development, for example when concrete spatial domains are mapped onto abstract temporal ones (Lakoff & Johnson, 1980).

This trajectory matches long-standing developmental evidence showing that children struggle to comprehend the psychological and metaphorical meanings of dual-function terms until they have firmly anchored their underlying physical meanings (Asch & Nerlove, 1960; Keil, 1986). Ultimately, these patterns suggest that the semantic architecture of early input is not merely a simplified version of adult communication, but a structured developmental bridge that enables the child’s mind to progressively build abstract thought out of concrete experience.

### Limitations and Future Directions

While the present study provides a large-scale, quantitative assessment of semantic constraints in child-directed speech, several methodological and corpus-specific limitations must be acknowledged.

First, our computational approach to figurative language relied on a binary classification paradigm via the MelBERT framework. In natural language processing and cognitive linguistics, metaphoricity is widely understood to be gradient rather than discrete, existing along a continuum from highly novel, salient extensions to deeply entrenched, dead metaphors that may function as literalized expressions in everyday usage (Steen et al., 2010). A binary classification model inevitably collapses this nuance, potentially masking subtle stylistic variations in how caregivers deploy conventionalized versus creative nonliteral language.

Second, the structural scope of our automated and human annotations was strictly confined to token-level metaphoricity. Because the underlying model evaluates language at the unit of individual words to align with the Metaphor Identification Procedure, it inevitably flattens holistic, macro-level figurative phenomena—such as multi-word idioms, phrasal verbs, and extended discourse-level allegories—into disconnected single-token components. Rather than structurally excluding multi-word expressions, this token-level architecture deconstructs them, parsing individual constituent items instead of evaluating the unified idiom as a single semantic chunk. This boundary is particularly consequential when analyzing conversational registers like CDS. Caregiver–child interactions are highly formulaic and frequently rely on holistic, multi-word figurative routines or playful personifications embedded across turn-taking sequences (Gaskins & Rundblad, 2025). Processing these patterns through an isolated single-word lens risks underrepresenting the true density and structural cohesion of figurative framing available in early language environments.

Third, our human interrater reliability metrics demonstrated a noticeable register-based divergence, yielding a modest agreement index for child-directed speech (*κ* = 0.33) compared to the moderate-to-substantial alignment observed in adult registers. This discrepancy highlights the difficulty of annotating naturalistic spoken interaction within developmental contexts. Caregiver utterances are inherently fragmentary, characterized by low mean length of utterance (MLU), frequent ellipsis, and heavy reliance on the immediate physical environment for deixis and reference grounding. Without an exhaustive multimodal record of the physical baseline (such as video tracking of joint attention and gaze), isolated transcriptions of short utterances introduce systematic ambiguity, making it difficult for both human coders and computational architectures to definitively isolate literal intent from nonliteral extension.

Fourth, important sampling constraints limit the generalizability of our empirical findings. Our analyses relied entirely on English-language corpora captured from specific socioeconomic Western populations. Culturally specific communicative styles, distinct patterns of caregiver scaffolding, and systemic structural properties of individual languages heavily influence how caretakers modify their speech. Future cross-linguistic and cross-cultural research is essential to verify whether parallel semantic adaptations—specifically the targeted suppression of metaphoricity and elevation of sensory-grounded terms—characterize CDS universally, or whether they reflect localized, culturally specific pedagogical ideologies.

Finally, it must be emphasized that direct, dyadic caregiver interaction constitutes only a partial component of a child’s total linguistic environment. Children do not acquire language from child-directed input alone; rather, they are continuously exposed to a heterogeneous mix of speech registers, including peer-to-peer play, sibling interactions, and dense streams of overheard, multi-party adult conversations that are not structurally or semantically tailored to the child’s processing limits (Bunce et al., 2025; Shneidman & Goldin-Meadow, 2012). Because the present investigation focused exclusively on the child-directed register within the available English corpus, future research should compare these findings with caregiver input from parents and communities in which highly modified, dyadic CDS is less frequent or less culturally normative. Such comparisons would make it possible to test whether the reduced metaphoricity and elevated concreteness observed here are general properties of speech addressed to children, or whether they are specifically associated with standard, highly scaffolded CDS. They would also help establish whether children exposed to less modified input encounter a semantic environment closer to adult-directed speech, with potentially greater figurative density, lower concreteness, or weaker register separation. Future research must therefore systematically expand these automated semantic mapping techniques to alternative registers within the broader input environment to capture the true ecological baseline of language acquisition.

### Conclusion

Despite these limitations, the present study demonstrates that the complex semantic architecture of child-directed speech—long assumed in developmental theory but rarely subjected to large-scale computational evaluation—can be systematically quantified. By coupling state-of-the-art metaphor detection with multi-dimensional human concreteness norms, this work provides empirical evidence that caregiver input is systematically structured along semantic dimensions that prioritize grounded, repetitive, and semantically transparent language. This protective configuration reduces referential ambiguity during the fragile initial stages of word mapping. As the child develops, this semantic scaffolding is gradually relaxed, supporting a gradual, age-dependent transition from literal, sensory-anchored communication to the abstract spaces of mature human lexical creativity.

## FUNDING INFORMATION

This work was carried out within the framework of the National Centre of Competence in Research (NCCR) Evolving Language, supported by the Swiss National Science Foundation (SNSF) under grant numbers 180888 and 225146. Additional funding was provided by the Gerda Zeltner Fund at the University of Zurich. The authors also acknowledge institutional support from the Institute for the Interdisciplinary Study of Language Evolution (ISLE), University of Zurich.

## CODE AND DATA AVAILABILITY STATEMENT

All code and data associated with this study are available at https://github.com/lingresearchers/LexCreativity. The repository contains the analysis scripts, processed datasets, and documentation required to reproduce the reported results.

## Note

^1^ After our initial experiments with MelBERT, we also conducted exploratory in-context learning studies with LLMs such as Gemini and GPT-4o to assess whether purely pretrained models might produce annotations that better aligned with human judgments. These experiments did not yield substantially better results. Nonetheless, the full potential of in-context learning—particularly with long-context inputs that allow the inclusion of the entire MIP instruction manual, and the possibility of moving beyond binary classification to identify conceptual mappings or generate explicit classification rationales—remains promising, but is beyond the scope of this paper.
